# Persistently Active *Helicobacter pylori* Infection Is Associated with the Development of Metabolic Dysfunction-Associated Steatotic Liver Disease

**DOI:** 10.3390/jcm14041073

**Published:** 2025-02-07

**Authors:** Jun Young Kim, Byung Soo Kwan, Jung Hwan Cho, Hye In Kim, Nak Gyeong Ko, Mihyeon Jin, Ok Jae Lee

**Affiliations:** 1 Department of Medicine, Gyeongsang National University Graduate School, Jinju 52727, Republic of Korea; jyk2412@gmail.com; 2 Department of Internal Medicine, Samsung Changwon Hospital, Sungkyunkwan University School of Medicine, Changwon 51353, Republic of Korea; 3 Department of Research & Support, Samsung Changwon Hospital, Sungkyunkwan University School of Medicine, Changwon 51353, Republic of Korea; 4 Department of Internal Medicine, Gyeongsang National University College of Medicine and Gyeongsang National University Hospital, Jinju 52727, Republic of Korea; 5 Institute of Medical Science, Gyeongsang National University, Jinju 52727, Republic of Korea

**Keywords:** *Helicobacter pylori*, metabolic dysfunction-associated steatotic liver disease, non-alcoholic fatty liver disease, cohort study

## Abstract

**Background/Objectives:** Previous studies suggested a link between *Helicobacter pylori* (*H. pylori)* infection and steatotic liver disease, now termed metabolic dysfunction-associated steatotic liver disease (MASLD). This study aimed to identify the association of active *H. pylori* infection and the new concept of MASLD in a longitudinal cohort. **Methods:** We reviewed 1497 health examinees who had two endoscopic biopsies for *H. pylori* activity without hepatic steatosis at the baseline abdominal ultrasonography. Subjects were classified into four groups based on *H. pylori* activity. Multivariable Cox models assessed the link between active *H. pylori* infection status and incident MASLD. **Results:** Over a median follow-up of 31.1 months, 247 subjects (16.5%) developed MASLD. The groups were: *H. pylori* naïve (n = 57, 15.6%), de novo (n = 31, 15.3%), eradicated (n = 32, 16.1%), and persistent (n = 127, 17.4%). The *H. pylori* persistent group had a higher risk of MASLD compared to naïve group (hazard ratio: 1.41; 95% confidence interval: 1.01–1.96; *p*-value = 0.045). The association between *H. pylori* infection and incident MASLD was significant only with ongoing infection. **Conclusions:** Persistent *H. pylori* infection increases the risk of MASLD, indicating that active infection may contribute to MASLD development. Eradicating active *H. pylori* infection might help lower the incidence of MASLD.

## 1. Introduction

*Helicobacter pylori* (*H. pylori*) is a bacterium that colonizes the gastric environment. It is one of the most common chronic bacterial infections in humans, with about two thirds of the world’s general population currently infected [1,2]. It is well established that *H. pylori* infection is related to the development of intra-gastric diseases including chronic gastritis, peptic ulcer, mucosa-associated lymphoid tissue lymphoma, and gastric adenocarcinoma [3]. Beyond its established local pathologies, several pathophysiologic mechanisms linked to *H. pylori* infection, including chronic inflammation, alteration of gut microbiome, and malabsorption of nutrients, have been thought to result in extra-gastric systematic manifestations [4]. Since the 1980s, studies have found that this infection is associated with extra-gastric manifestations such as metabolic syndrome, diabetes, coronary heart diseases, liver tumor, and steatotic liver disease, previously known as non-alcoholic fatty liver disease (NAFLD) [5,6,7,8].

Steatotic liver disease is characterized by hepatic steatosis. It is a leading cause of chronic liver disease with a high global prevalence [9]. In 2023, more than 200 experts in liver disease formalized to rename NAFLD to a metabolic dysfunction-associated steatotic liver disease (MASLD) [10]. MASLD is diagnosed when cardiometabolic risk factors coexisted and have been suggested to identify more significant fibrosis than NAFLD, though most of the NAFLD meet MASLD criteria [10,11,12].

Development of MASLD involves a multiple-hit mechanism of abnormal lipid metabolism, insulin resistance with environmental exposure, and genetic susceptibility [13]. Studies have suggested that dysbacteriosis of the intestinal flora including *H. pylori* can induce abnormal lipid metabolism in the liver, eventually leading to the development of steatotic liver disease [14,15,16]. Recently, studies including meta-analysis have revealed that *H. pylori* infection is associated with MASLD development and that *H. pylori* eradication could reduce the risk MASLD [17,18,19,20]. Controversially, there are continuous debates on this subject, with some studies suggesting that *H. pylori* is not associated with MASLD [21,22,23,24]. Previous studies had limitations in that study types were mostly cross-sectional, with diagnosis of *H. pylori* performed by non-invasive tests which could not represent an active infection status. Therefore, this study aimed to identify the association of active *H. pylori* infection diagnosed by serial invasive tests and the risk of MASLD in a cohort of asymptomatic subjects with a longitudinal follow-up.

## 2. Materials and Methods

### 2.1. Database

**Figure 1:**
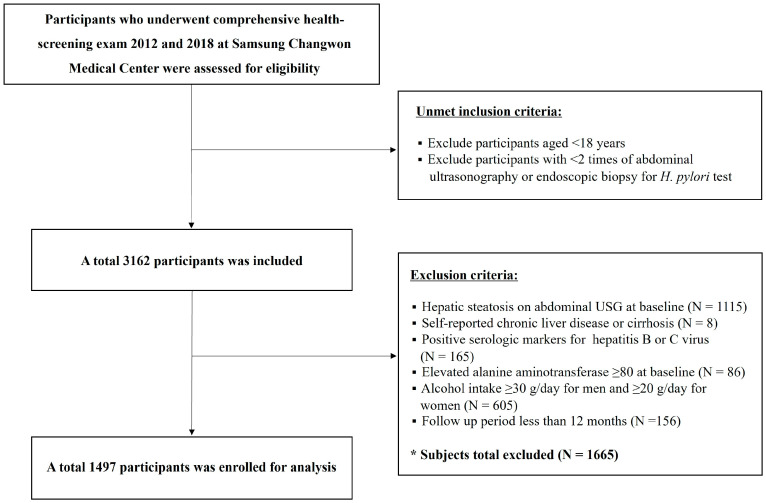
Flowchart of participant enrollment. *H. pylori*, *Helicobacter pylori*; USG, ultrasonography.

This was a retrospective cohort study performed at Samsung Changwon Medical Center, South Korea. The flowchart for study selection is shown in Figure 1. We screened healthy adults aged 19 years or older in a health screening cohort who participated in a comprehensive health-screening exam at the center of Samsung Changwon Medical Center between January 2012 and December 2018. Since we aimed to find out the longitudinal effect of active *H. pylori* infection status on the development of MASLD, we included participants who underwent invasive diagnostic test of *H. pylori* twice on endoscopy with at least two abdominal ultrasounds taken (the second endoscopy was performed at least 1 year apart from the baseline). We excluded subjects who had hepatic steatosis on baseline ultrasound (n = 1115) and possible specific etiology for steatotic liver diseases such as history of chronic liver disease (positive serologic test for hepatitis B or C virus or cirrhosis) (n = 8), elevated alanine aminotransferase ≥ 80 U/I (n = 86), and alcohol intake ≥ 30 g/day for men and ≥20 g/day for women (n = 605). Finally, a total of 1497 participants were included in the analysis. This study was conducted in accordance with the Declaration of Helsinki. The study protocol was approved by the Institutional Review Board (IRB) of Samsung Changwon Medical Center on 20 August 2023 (IRB approval No. SCMC 2023-08-005). The requirement to obtain informed patient consent was waived by the IRB because this study was retrospective by analyzing existing administrative and clinical data.

### 2.2. Data Collection and Variables

Clinical variables collected were demographic characteristics (including age, sex and body mass index (BMI)), detailed physical examination including systolic blood pressure (SBP), and a self-administered health questionnaire on smoking, alcohol consumption, physical activity, medication use, and medical history (including hypertension, diabetes mellitus, and dyslipidemia). Serum biochemical markers including fasting glucose, high-sensitivity C-reactive protein (hsCRP), lipid profile (such as total cholesterol (TC), triglycerides, low-density lipoprotein cholesterol (LDL), and high-density lipoprotein cholesterol (HDL)), and liver enzyme profile (such as aspartate aminotransferase (AST), alanine aminotransferase (ALT), and gamma-glutamyl transferase (GGT)) were measured after more than 12 h of fasting by the automated chemistry analyzer AU5822 (Beckman Coulter, Brea, CA, USA) and reviewed from electronic medical records.

BMI was calculated as weight in kilograms/height in square meters (kg/m^2^) and categorized as underweight (BMI < 18.5), normal (18.5 ≤ BMI < 24.9), overweight (25.0 ≤ BMI < 29.9), and obese (BMI ≥ 30.0). Smoking status was categorized as currently non-smoker, moderate smoker (<1 pack/day), and heavy smoker (≥1 pack/day). Alcohol consumption was divided into mild (<10 g/day) and modest (≥10 g/day) (A standard drink is defined as a 10 g/day of alcohol consumption by the guidelines in The Alcohol Use Disorders Identification Test by World Health Organization). Regular exercise was determined when moderate to hard intense physical activity was performed three or more times per week. Dyslipidemia was defined when one or more lipid profiles met the definition of criteria (HDL ≤ 40 mg/dL or TG ≥ 200 mg/dL or LDL ≥ 160 mg/dL) or currently on dyslipidemia medication in a questionnaire [25].

### 2.3. H. pylori Diagnosis and Definition of Active H. pylori Infection Status

During the upper endoscopy exam, biopsies were performed for the *H. pylori* special stain (Warthin–Starry stain) or rapid urease test (Pyloplus, ARJ Medical Inc, Oldsmar, FL, USA) on at least two sites of the stomach of greater curvature sides of the antrum and body. Tests were performed for participants who voluntarily wanted the *H. pylori* test or were suspected to have active *H. pylori* gastritis, exhibiting symptoms such as diffuse redness, enlarged folds, mucosal edema, and nodularity [26].

**Figure 2:**
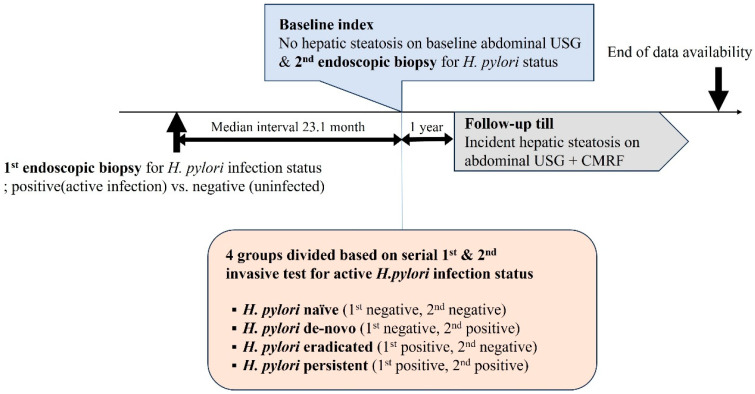
Schematic diagram of the study design. *H. pylori*, *Helicobacter pylori*; USG, ultrasonography; CMRF, cardiometabolic risk factor.

As we wanted to know the impact of ongoing activity of *H. pylori* infection on incident of MASLD, participants were divided into 4 groups based on the result of serial invasive diagnostic tests: (1) *H. pylori* naïve group, when active *H. pylori* infection was not identified on the two continuous tests; (2) *H. pylori* de novo group, when initial *H. pylori* negative infection state on the first exam turned up to be positive on the follow up study; (3) *H. pylori* eradicated group, when a positive infection state turned up to be negative; and (4) *H. pylori* persistent group, when sustained active *H. pylori* infection was detected during serial exams. A schematic diagram of the study design is shown in Figure 2.

### 2.4. Definition of MASLD Development

The primary outcome was MASLD development during follow-up. Hepatic steatosis was defined by abdominal ultrasonography based on standard criteria such as diffuse increased echogenicity of liver parenchyma, deep attenuation, hepatorenal echo contrast, and vessel blurring [27]. MASLD was diagnosed when at least one or more of the following five cardiometabolic risk factors were detected at the time of incident hepatic steatosis: (1) BMI ≥ 23 kg/m^2^ or waist circumference > 90 cm for males or 80 cm for females; (2) fasting serum glucose ≥ 100 mg/dL or 2 h post-load glucose levels ≥ 140 mg/dL or HbA1 c ≥ 5.7% or diagnosis of type 2 diabetes or treatment for type 2 diabetes; (3) blood pressure ≥ 130/85 mmHg or specific antihypertensive drug treatment; (4) plasma triglycerides ≥ 150 mg/dL or lipid-lowering treatment; (5) plasma HDL ≤ 40 mg/dL for males or ≤50 mg/dL for females or lipid-lowering treatment [10].

We excluded subjects with possible specific etiology for steatotic liver disease such as excessive alcohol consumption, chronic liver disease including viral hepatitis, and elevated ALT over 2 folds of upper normal limit at baseline. Therefore, cases of newly found hepatic steatosis with cardiometabolic risk factor were considered incident MASLD.

### 2.5. Statistical Analysis

Continuous variables were compared using a Student’s *t*-test, and categorical variables were compared using one-way ANOVA, Kruskal–Wallis test, or Pearson’s chi-square test. Descriptive statistics were used to summarize participants’ baseline characteristics between groups and post hoc analysis was performed using Bonferroni’s adjustment.

The primary endpoint was incident MASLD. Cox proportional hazard models were used. Multivariable models were analyzed after adjusting for age, sex (Model 1), and metabolic risk factors such as BMI, exercise, smoking, drinking, blood pressure, fasting glucose, and presence of dyslipidemia (Model 2). Liver enzyme profiles of AST, ALT, GGT were further adjusted (Model 3). Statistical significance was considered when the *p*-value was less than 0.05. All statistical analyses were performed using SAS software version 9.4 (SAS Institute, Cary, NC, USA) and IBM SPSS version 25.0 (SPSS Inc., IBM Corporation, Chicago, IL, USA).

## 3. Results

### 3.1. Basic Characteristics of the Study Cohort

Table 1 shows demographic and clinical characteristics of the 1497 included participants. The mean age of the study population was 46.7 years. There were 956 (63.9%) males. According to the active *H. pylori* infection status, *H. pylori* naïve, *H. pylori* de novo, *H. pylori* eradicated, and *H. pylori* persistent groups had 365, 203, 199, and 730 subjects, respectively. The median interval between two serial endoscopy exams was 23.1 months. There were statistically significant differences in age, regular exercise, systolic blood pressure, and AST between groups. During follow-up, MASLD developed in 247 (16.5%) cases in the total study population (n = 1497). The median follow-up period was 31.1 months (rage, 12 to 83.3 months). The percentage of incident MASLD was 15.6% in the *H. pylori* naïve group, 15.3% in the *H. pylori* de novo, 16.1% in the *H. pylori* eradicated group, and 17.4% in the *H. pylori* persistent group.

### 3.2. Factors Associated with MASLD Development

**Figure 3:**
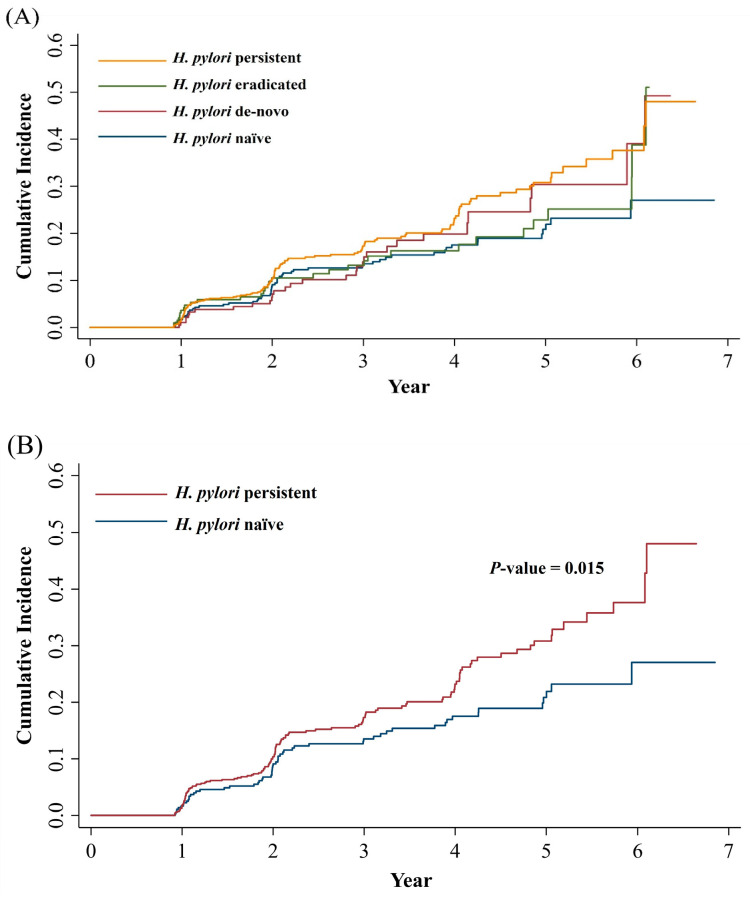
(**A**) Cumulative incidence for MASLD by active *H. pylori* infection status divided by 4 groups. (**B**) Cumulative incidence for MASLD by *H. pylori* naïve and persistent infection (*p*-value = 0.015, log rank test). MASLD, metabolic dysfunction-associated steatotic liver disease; *H. pylori*, *Helicobacter pylori*.

Compared to the *H. pylori* naïve group, the *H. pylori* persistent group showed a higher risk of MASLD development in univariable and multivariable Cox proportional hazard models (univariable hazard ratio [HR]: 1.48; 95% confidence interval [CI]: 1.08–2.02; *p* = 0.015; multivariable HR: 1.41; 95% CI: 1.01–1.96; *p* = 0.045). Other than active *H. pylori* infection status, age, male sex, higher BMI, fasting glucose, ALT, GGT, and presence of dyslipidemia were also significant factors associated with MASLD development in multivariable analysis (Table 2). We used serial multivariable adjustment models to figure out the interaction between active *H. pylori* infection and such factors on the development of MASLD. Model 1 adjusted for age and sex, Model 2 adjusted for additional metabolic risk factors (BMI, exercise, smoking, alcohol drinking, SBP, fasting glucose, and dyslipidemia), and, finally, Model 3 was adjusted for additional liver function profiles (AST, ALT and GGT). The *H. pylori* persistent group showed a higher risk for MASLD development than the *H. pylori* naïve group in multivariable Model 1 and final multivariable Model 3, but not statistically significant in Model 2 (HR: 1.39; 95% CI: 1.00–1.92; *p* = 0.051) (Table 3). Figure 3 shows cumulative incidence of MASLD based on active *H. pylori* infection status.

### 3.3. Active H. pylori Infection and the Development of MASLD

**Figure 4:**
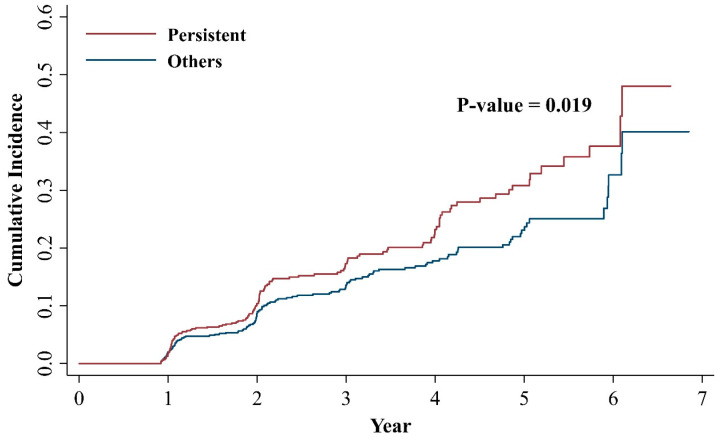
Cumulative incidence for MASLD by *H. pylori* persistent infection and others. MASLD, metabolic dysfunction-associated steatotic liver disease; *H. pylori*, *Helicobacter pylori*.

We further analyzed the association between various *H. pylori* infection status and the MASLD development. Results are shown in Table 4. When participants were divided by the last active *H. pylori* infection status (i.e., the presence of *H. pylori* infection at the baseline index regardless of previous infection), the association between the last active *H. pylori* infection and incident MASLD was attenuated after serial adjustments. However, when the *H. pylori* persistent group was compared with rest of others, the association between persistent *H. pylori* infection and MASLD development was statistically significant in all multivariable analyses (Model 1, 2, and Model 3 (HR: 1.33; 95% CI: 1.02–1.73; *p* = 0.033)). Results of cumulative incidence of MASLD in the *H. pylori* persistent group and others are shown in Figure 4.

## 4. Discussion

In this cohort study, we found that participants with persistently active *H. pylori* infection were at a higher risk of MASLD than those with infection-naïve or other infection statuses. Other risk factors for incident MASLD included age, male gender, higher BMI, fasting glucose, ALT, and GGT.

We focused on the diagnosis of active *H. pylori* infection by serial invasive histologic biopsies which guarantee a high diagnostic specificity. A serial biopsy results of infection status meant that active infection had persisted at least during the interval between two biopsies and the *H. pylori* persistent group was assumed to have the highest exposure to an active infection. Our findings indicate that continuous exposure to active *H. pylori* infection contributed to MASLD pathogenesis. We also observed an increasing trend, although not statistically significant, between *H. pylori* naïve group and the eradicated or de novo group. This implies that eradication or prevention of the active infection might lower the incidence of MASLD, although more study is required.

Previous studies, including high-quality meta-analyses, have demonstrated an association between MASLD and *H. pylori* infection. Although heterogeneity existed according to sample size, study method, and the method of diagnosing *H. pylori* infection, meta-analyses studies have shown high odds ratios of MASLD ranging from 1.19 to 1.27 in *H. pylori* positive patients compared to *H. pylori* negative patients [19,28,29,30]. A recent large population-based study has also shown high odds ratio of 2.51 for metabolic dysfunction-associated steatohepatitis, a severe form of MASLD in *H. pylori* infection [31]. However, some unmet needs existed in previous studies because most studies had a cross-sectional, or case–control design and non-invasive tests were mostly used to evaluate *H. pylori* infection. A prospective cohort study with a large study population of 17,028 South Korean adults followed for over 81,130 person-years has found a higher incidence of MASLD in *H. pylori*-seropositive patients [17]. As the authors mentioned in study limitations, seropositivity for *H. pylori* diagnosis was evaluated in their study, which had a limited effectiveness because diagnostic accuracy might be low. In addition, their study included all patients with past, eradicated, and present *H. pylori* infection. Therefore, it could not accurately distinguish active infection. One retrospective study of 64 obese patients undergoing bariatric surgery has demonstrated that active *H. pylori* infection is independently associated with biopsy-proven non-alcoholic steatohepatitis and fibrosis [32]. A few non-longitudinal studies have used invasive histologic tests to confirm the diagnosis of *H. pylori* infection [33,34,35,36]. In comparison with these studies which used non-invasive diagnostic modality or non-longitudinal studies, our study has strengths in the study methodology.

Many theories have been suggested about the relationship between *H. pylori* and MASLD, although more detailed studies are needed. Possible contributions of *H. pylori* infection to MASLD development include chronic inflammation, altered gut microbiota, increased intestinal permeability, increased insulin resistance, dyslipidemia, and metabolic syndrome, and so on [37,38,39]. Pro-inflammatory cytokines increased by *H. pylori* infection can generate low grade systemic inflammation. When the gastrointestinal epithelium is disrupted by *H. pylori*, infection related metabolites are absorbed through portal flow to the liver [40]. Finally, toll-like receptors can activate the inflammatory process and lead to hepatic steatosis [41]. *H. pylori* infection has also been suggested to be associated with metabolic syndrome by several possible potential mechanisms in meta-analysis and systematic reviews [8,42,43]. Metabolic syndrome and MASLD are also strongly associated in both directions [44]. In our study, a statistically significant association between *H. pylori* infection and MASLD was attenuated when metabolic variables were adjusted in Model 2 analysis. This might be due to a confounding effect of *H. pylori*, metabolic dysregulation, and MASLD development. From these findings, potential pathogenetic mechanisms between *H. pylori* infection and metabolic dysregulation may be hypothesized, eventually resulting in the development of MASLD.

Although this study is strengthened by a cohort study design and diagnostic methods of active *H. pylori* based on serial invasive endoscopic biopsies, we should acknowledge that our present study has several limitations. First, the retrospective design of this study inherits potential selection, measurement, and recall biases. We included participants who had no specific symptoms based on a questionnaire. However, possible symptomatic participants, such as those with gastroesophageal reflux, are more likely to undergo endoscopic exams. Given that obesity is strongly associated with gastroesophageal reflux and MASLD, participants who underwent endoscopy were more likely to have higher BMI than those without undergoing an upper endoscopy. By this selection, the association between *H. pylori* and MASLD might have been inflated. Second, hepatic steatosis was defined by abdominal ultrasonography known to have a wide range of sensitivity and specificity [45]. Finally, the study population consisted of mostly healthy Asian individuals in a single center.

## 5. Conclusions

We report that a persistently active *H. pylori* infection is associated with a higher risk of MASLD development. To the best of our knowledge, this was the first longitudinal study that evaluated the association between *H. pylori* infection and the risk of MASLD based on active infection diagnosed by serial invasive biopsies (i.e., the gold standard method). Our study provides new insights into the current field of the relationship between *H. pylori* and MASLD and, if verified, eradication therapy for asymptomatic individuals with active infection may yield benefits by reducing the risk of MASLD. Future studies including other populations in multiple centers and randomized controlled trials are needed to evaluate if bacterial elimination can have a consistent therapeutic effect in reducing the incidence of MASLD and eventually reducing related comorbidities.

## Figures and Tables

**Table 1:** Baseline characteristics of the study population.

	Total (n = 1497)	*H. pylori* Naïve (n = 365)	*H. pylori* De Novo (n = 203)	*H. pylori* Eradicated (n = 199)	*H. pylori* Persistent (n = 730)	*p*-Value
Age (years)	46.71 ± 7.17	48.85 ± 7.67	47.83 ± 7.14	46.72 ± 7.29	45.32 ± 6.56	<0.001
Male (%)	956 (63.9)	218 (59.7)	133 (65.5)	133 (66.8)	472 (64.7)	0.272
Body mass index (kg/m^2^)	23.14 ± 2.55	23.02 ± 2.42	23.18 ± 2.58	23.14 ± 2.40	23.20 ± 2.65	0.740
<18.5	44 (2.94)	10 (2.74)	5 (2.46)	5 (2.51)	24 (3.29)	
18.5–24.9	1110 (74.15)	278 (76.16)	145 (71.43)	150 (75.38)	537 (73.56)	
25–29.9	305 (20.37)	66 (18.08)	46 (22.66)	43 (21.61)	150 (20.55)	
≥30	17 (1.14)	6 (1.64)	1 (0.49)	0 (0.00)	10 (1.37)	
Smoking						0.296
Never smoker	533 (35.60)	125 (34.25)	68 (33.50)	77 (38.69)	263 (36.03)	
Current(<1 pack/day)	428 (28.59)	97 (26.58)	59 (29.06)	56 (28.14)	216 (29.59)	
Current(≥1 pack/day)	268 (17.90)	59 (16.16)	38 (18.72)	37 (18.59)	134 (18.36)	
Alcohol						0.085
Mild (<10 g/day)	442 (29.53)	116 (31.78)	53 (26.11)	55 (27.64)	218 (29.86)	
Modest (≥10 g/day)	690 (46.09)	151 (41.37)	89 (43.84)	103 (51.76)	347 (47.53)	
Regular exercise	229 (19.97)	74 (20.27)	34 (16.75)	44 (22.11)	147 (20.14)	0.021
hsCRP (mg/dL)	1.02 ± 3.29	0.96 ± 2.28	1.47 ± 5.72	1.02 ± 2.19	0.93 ± 3.04	0.222
SBP (mmHg)	116.66 ± 11.38	116.31 ± 11.65	118.79 ± 11.58	117.94 ± 10.98	115.90 ± 11.22	0.005
Fasting glucose (mg/dL)	89.46 ± 14.74	89.46 ± 11.69	89.58 ± 21.77	89.97 ± 18.96	89.28 ± 12.22	0.950
AST (U/I)	22.11 ± 8.37	23.30 ± 9.16	22.34 ± 8.73	22.98 ± 9.46	21.21 ± 7.41	<0.001
ALT (U/I)	20.29 ± 10.37	20.61 ± 10.46	20.79 ± 10.40	20.87 ± 10.96	19.83 ± 10.14	0.411
GGT (U/I)	26.55 ± 28.65	24.57 ± 22.89	27.71 ± 28.58	24.68 ± 21.93	27.74 ± 32.58	0.247
Dyslipidemia (%)	27.5	26.8	29.1	25.1	28.1	0.798
HOMA-IR	0.94 ± 0.61	0.92 ± 0.57	0.92 ± 0.66	0.95 ± 0.59	0.95 ± 0.62	0.925
MASLD (%)	247 (16.5)	57 (15.6)	31 (15.3)	32 (16.1)	127 (17.4)	
Follow up (month)	31.1 (12, 83.3)	39.0 (12, 83.3)	33.8 (12, 77.5)	31.9 (12, 74.6)	25.5 (12, 80.8)	
Interval between serial biopsy (months)	23.1 (5.0, 77.5)	21.6 (5.0, 73.1)	24.6 (7.4, 70.3)	22.4 (5.2, 73.7)	23.2 (5.8, 77.5)	

Values are presented as mean ± standard deviation, median (min, max) or number (%) by descriptive analysis, and frequency analysis. *H. pylori*, *Helicobacter pylori*; hsCRP, high-sensitivity C-reactive protein: SBP, systolic blood pressure: AST, aspartate aminotransferase; ALT, alanine aminotransferase; GGT, gamma-glutamyltransferase; HOMA-IR, homeostasis model assessment of insulin resistance; MASLD, metabolic dysfunction-associated steatotic liver disease. Dyslipidemia: HDL ≤ 40 mg/dL or TG ≥ 200 mg/dL or LDL ≥ 160 mg/dL or use of dyslipidemia medication.

**Table 2:** Factors associated with MASLD development.

	Univariable Analysis		Multivariable Analysis	
	HR (95% CI)	*p*-Value	HR (95% CI)	*p*-Value
Age	1.02 (1.00, 1.04)	0.019	1.02 (1.00, 1.04)	0.033
Male sex	3.00 (2.14, 4.21)	<0.001	1.89 (1.18, 3.01)	0.008
Body mass index (kg/m^2^)				
<18.5	-		-	
18.5–24.9	Reference		Reference	
25–29.9	2.87 (2.20, 3.74)	<0.001	1.99 (1.49, 2.66)	<0.001
≥30	6.70 (3.52, 12.75)	<0.001	6.48 (3.31, 12.69)	<0.001
Smoking				
Never smoker	Reference		Reference	
Current(<1 pack/day)	2.50 (1.76, 3.56)	<0.001	1.40 (0.92, 2.13)	0.115
Current(≥1 pack/day)	2.47 (1.67, 3.65)	<0.001	1.17 (0.75, 1.84)	0.487
Alcohol				
Mild (<10 g/day)	Reference		Reference	
Modest (≥10 g/day)	1.64 (1.19, 2.26)	0.002	0.91 (0.64, 1.29)	0.598
Regular exercise	0.84 (0.61, 1.17)	0.315	0.83 (0.59, 1.17)	0.288
hsCRP (mg/dL)	1.02 (1.00, 1.05)	0.074		
SBP (mmHg)	1.02 (1.01, 1.03)	0.001	1.00 (0.99, 1.01)	0.726
Fasting glucose (mg/dL)	1.01 (1.01, 1.02)	<0.001	1.01 (1.00, 1.01)	0.022
AST (U/I)	1.02 (1.01, 1.04)	<0.001	0.99 (0.97, 1.01)	0.406
ALT (U/I)	1.04 (1.03, 1.05)	<0.001	1.02 (1.01, 1.04)	0.004
GGT (U/I)	1.01 (1.01, 1.01)	<0.001	1.01 (1.00, 1.01)	0.003
Dyslipidemia (%)	2.23 (1.73, 2.86)	<0.001	1.51 (1.15, 1.97)	0.003
Active *H. pylori* infection				
*H. pylori* naïve	Reference		Reference	
*H. pylori* de novo	1.21 (0.78, 1.88)	0.388	1.04 (0.65, 1.65)	0.884
*H. pylori* eradicated	1.18 (0.77, 1.82)	0.446	1.18 (0.75, 1.85)	0.472
*H. pylori* persistent	1.48 (1.08, 2.02)	0.015	1.41 (1.01, 1.96)	0.045

*p*-values were calculated using the Cox proportional hazard model. MASLD, metabolic dysfunction-associated steatotic liver disease; HR, hazard ratio; CI, confidence interval; *H. pylori, Helicobacter pylori*; hsCRP, high-sensitivity C-reactive protein; SBP, systolic blood pressure; AST, aspartate aminotransferase; ALT, alanine aminotransferase; GGT, gamma-glutamyltransferase. Dyslipidemia: HDL ≤ 40 mg/dL or TG ≥ 200 mg/dL or LDL ≥ 160 mg/dL or use of dyslipidemia medication.

**Table 3:** Serial multivariable adjustment models of active *H. pylori* infection and incident MASLD.

	Model 0		Model 1		Model 2		Model 3	
Adjusted for	-		Age, Sex		Metabolic Risk		Liver Profiles	
	HR (95% CI)	*p*-Value	HR (95% CI)	*p*-Value	HR (95% CI)	*p*-Value	HR (95% CI)	*p*-Value
Naïve (−/−)	Reference		Reference		Reference		Reference	
De novo (−/+)	1.21 (0.78, 1.88)	0.388	1.13 (0.73, 1.76)	0.573	1.11 (0.70, 1.76)	0.659	1.04 (0.65, 1.65)	0.884
Eradicated (+/−)	1.18 (0.77, 1.82)	0.446	1.16 (0.75, 1.79)	0.516	1.13 (0.72, 1.76)	0.596	1.18 (0.75, 1.85)	0.472
Persistent (+/+)	1.48 (1.08, 2.02)	0.015	1.48 (1.07, 2.04)	0.017	1.39 (1.00, 1.92)	0.051	1.41 (1.01, 1.96)	0.045

*p*-values were calculated using the Cox proportional hazard model. Model 1: Adjusted for age, sex. Model 2: Model 1 plus adjustment for BMI, exercise, smoking, alcohol drinking, SBP, fasting glucose, dyslipidemia. Model 3: Model 2 plus adjustment for AST, ALT, GGT. *H. pylori*, *Helicobacter pylori*; MASLD, metabolic dysfunction-associated steatotic liver disease; HR, hazard ratio; CI, confidence interval; BMI, body mass index; SBP, systolic blood pressure; AST, aspartate aminotransferase; ALT, alanine aminotransferase; GGT, gamma-glutamyltransferase. Dyslipidemia: HDL ≤ 40 mg/dL or TG ≥ 200 mg/dL or LDL ≥ 160 mg/dL or use of dyslipidemia medication.

**Table 4:** Active *H. pylori* infection and the development of MASLD.

	Model 0		Model 1		Model 2		Model 3	
Adjusted for	-		Age, Sex		Metabolic Risk		Liver Profiles	
	HR (95% CI)	*p*-Value	HR (95% CI)	*p*-Value	HR (95% CI)	*p*-Value	HR (95% CI)	*p*-Value
Last active *H. pylori* infection (*H. pylori* infection at baseline index)								
*H. pylori* (−)	Reference		Reference		Reference		Reference	
*H. pylori* (+)	1.34 (1.03, 1.74)	0.029	1.32 (1.02, 1.73)	0.038	1.27 (0.97, 1.66)	0.086	1.24 (0.94, 1.63)	0.122
Persistently active *H. pylori* infection								
Others	Reference		Reference		Reference		Reference	
Persistent	1.35 (1.05, 1.73)	0.019	1.38 (1.07, 1.78)	0.014	1.31 (1.01, 1.69)	0.043	1.33 (1.02, 1.73)	0.033

*p*-values were calculated using the Cox proportional hazard model. Model 1: Adjusted for age, sex. Model 2: Model 1 plus adjustment for BMI, exercise, smoking, alcohol drinking, SBP, fasting glucose, dyslipidemia. Model 3: Model 2 plus adjustment for AST, ALT, GGT. *H. pylori, Helicobacter pylori*; MASLD, metabolic dysfunction-associated steatotic liver disease; HR, hazard ratio; CI, confidence interval; BMI, body mass index; SBP, systolic blood pressure; AST, aspartate aminotransferase; ALT, alanine aminotransferase; GGT, gamma-glutamyltransferase. Dyslipidemia: HDL ≤ 40 mg/dL or TG ≥ 200 mg/dL or LDL ≥ 160 mg/dL or use of dyslipidemia medication.

## Data Availability

The data that support the findings of this study are available from the corresponding author upon reasonable request.
